# Community pharmacists’ perceived value on precision medicine, desired training components, and exposure during pharmacy education: Malaysia’s experience

**DOI:** 10.3389/fphar.2022.978141

**Published:** 2022-09-21

**Authors:** Faiza Naimat, Mathumalar Loganathan Fahrni, Shankar Purushothaman, Mohamad Nizam Abdul Ghani, Supatat Chumnumwat, Zaheer-Ud-Din Babar

**Affiliations:** ^1^ Faculty of Pharmacy, Universiti Teknologi MARA (UiTM), Puncak Alam, Selangor, Malaysia; ^2^ School of Pharmacy, Management and Science University, Shah Alam, Malaysia; ^3^ Collaborative Drug Discovery Research (CDDR) Group, Communities of Research (Pharmaceutical and Life Sciences), Universiti Teknologi MARA (UiTM), Puncak Alam, Selangor, Malaysia; ^4^ Department of Pharmacy, Hospital Shah Alam, Shah Alam, Selangor, Malaysia; ^5^ Department of Pharmacy, Faculty of Pharmacy, Mahidol University, Bangkok, Thailand; ^6^ University of Huddersfield, Huddersfield, West Yorkshire, United Kingdom

**Keywords:** pharmacogenomics, pharmacogenetics, pharmacist, stakeholder, precision medicine, molecular diagnostics, personalised medicine

## Abstract

**Background:** Precision medicine beckons new horizons for therapy geared to one’s genetics, lifestyle, and environmental determinants. Molecular, pathology, and clinical diagnostics can be integrated to provide pharmaceutical care.

**Aims:** The value and appeal of precision medicine to community pharmacists, knowledge attained, and training programmes perceived as necessary were evaluated.

**Methods:** Over 10 months, a published questionnaire, which was also digitally accessible during the COVID-19 outbreak, was distributed by hand, *via* email and social media. 300 community pharmacists across 9 districts in an urban state in Malaysia, self-administered and returned completed versions (response rate 75%). Three- or five-point Likert scale and multiple-choice responses were analysed using SPSS to assess whether or not exposure through the pharmacy curricula impacted current knowledge, perception and willingness to pursue precision medicine.

**Results:** Respondents were largely: females (*N* = 196, 65.3%) and practicing for up to 10 years (*N* = 190, 66.3%). Although knowledge levels were moderate (76%), positive perceptions were showcased (94%), and 80% were willing to integrate precision medicine into their daily practice. Although 61% did not or do not recall having had prior exposure to pharmacogenomics as part of their pharmacy school curricula, many (93%) were willing to attain knowledge by undergoing additional training. Desired training included current pharmacogenetic testing available (17%), interpretation of the test results (15%), and ethical considerations (13%). Community pharmacists who had 0.5–10 years’ work experience possessed greater knowledge (μ = 1.48, CI 1.35–1.61, *p* = 0.017), than the pharmacists who had 21–40 years of work experience (μ = 1.28, CI 1.05–1.51, *p* = 0.021). Exposure to the subject during pharmacy education positively impacted the willingness to integrate precision medicine in daily practice (*p* = 0.035).

**Conclusion:** Community pharmacists were receptive to and valued precision medicine. A relatively high number had prior exposure to concepts of precision medicine through the pharmacy curriculum, and were therefore willing to adopt the practice in their day-to-day provision of healthcare. With adequate training centred on bioethics, utilising pharmacogenetic testing, and interpretation of the results, community pharmacists will be equipped for the provision of precision medicine services in the foreseeable future.

## 1 Introduction

The prescribing of precise and responsive pharmaceuticals matched to one’s genome sequence has been at the epicentre for accelerating positive patient outcomes globally. In the United Kingdom (Royal Pharmaceutical Society of Great Britain, 2022), a recent position statement, issued by the UK professional membership body for pharmacists and pharmacy, called for pharmacists to play a leading role in the fast-approaching pharmacogenomics revolution. This has shed more light on the benefits and application of genomic testing. Based on the Precision Medicine Initiative backed by a $215 million commitment by the United States of America’s administration in 2016 (Doll et al., 2021), precision medicine was defined as a transpiring proposition that adopted individual differences in genes, surroundings, and a person’s way of living for the alleviation of ailments and disease prevention (Collins et al., 2016). Precision medicine, similar to preventive healthcare, seeks to augment reactive medicine by pre-empting disease through preventive measures and early detection (Gambhir et al., 2018).

Therapies which are effective can be established for the specific patient and condition when the diagnosis is supplemented with a detailed genome analysis of the individual who is prone to the ailment. (Gambhir et al., 2018). To tailor the therapy to complement such a molecular level diagnosis for the specific patient, principles of prescribing which are termed precision, personalised, or individualised, have often been utilised interchangeably or in combination throughout the past decade. However, in modern times, the phrase “precision” instead of “personalised” is used liberally. Nevertheless, the common denominator for both precision and personalised medicine is its genomics (Ong et al., 2021).

Pharmacogenomics, a combination of pharmacology and genomics, is often touted to be at the core of precision medicine. Pharmacogenomics is coined as the study of genomic contribution as well as other “omics” to variations in drug reaction phenotype. These variations can include instances of less therapeutic effectiveness, as well as serious and fatal adverse drug reactions (Ong et al., 2021). Primary healthcare providers can utilise molecular understanding of patients’ genetics and subsequently recommend treatment regimens, thereby, enhancing their capability for patient selection. In addition, the prediction of successful therapeutic combinations can minimise adverse drug reactions (Yamamoto et al., 2022).

The revolution is likely to transform prescribing practices and patient outcomes by leaps and bounds. Pharmacists are uniquely positioned to lead optimal drug selection and dosing (Alkhoshaiban et al., 2019). The shared responsibilities with other healthcare providers such as physicians, laboratory scientists, and genetic counsellors, include, promoting the rational use of pharmacogenomic testing, interpreting the test results and communicating with patients, and with each other, optimising medication therapy based on test results and integrating the application of pharmacogenomics into platforms of clinical practice. (Papastergiou et al., 2017). Nevertheless, as it stands, there are marked disparities in the willingness to adopt and practice precision medicine (Elewa and Awaisu, 2019). As compared to the Western countries, particularly in the USA and Canada, where some use of therapeutic drug monitoring in combination with analysis of the liver and renal functions, genomics, and environmental and lifestyle exposure, as well as, other unique patient or disease characteristics were used to guide drug selection and dosing (Hicks et al., 2019), far fewer South East Asian studies of similar nature were evident.

Healthcare providers were neither willing, nor were they ready to implement precision medicine. The inherent hesitation to adopt the principle was largely due to the lack of infrastructure and guidance for genomics education. A study conducted in 2018 provided an impetus for dialogues to transpire among four South East Asian countries, namely Malaysia, Singapore, Thailand, and Indonesia. The study’s findings inferred that the practice of personalised medicine was more prevalent in Singapore and Thailand, but was lacking in Malaysia and Indonesia (Chong et al., 2018).

The aim of this research was to assess the value and appeal of precision medicine to community pharmacists, the knowledge attained, and their willingness to adopt the practice.

## 2 Materials and Methods

### 2.1 Study design and sampling

A population-based study of community pharmacists located in Malaysia’s west coast state of Selangor was conducted. Data was collected over 10 months, and across nine districts, from September 2019 to June 2020. The state of Selangor was identified as one of the areas in the nation with a high density of community pharmacies, and public health clinics, and a high hospital to population ratio. The existing legal framework provides the community pharmacists with the necessary empowerment to carry out community pharmacy practices. Of the nation’s 2,600 community pharmacies, the majority were located in Selangor and densely scattered in the Federal Territory Kuala Lumpur and its surroundings (Kho et al., 2018). All the community pharmacies were either funded independently or they operated as part of a supply chain (Kho et al., 2017).

Convenience sampling was used, where questionnaires were distributed to all pharmacists who fulfilled the study’s inclusion and exclusion criteria. Pharmacists who had been working for at least 6 months in a community-based pharmacy, which is registered with a postcode attached to any of the nine districts within the state were included. Both full-time and part-time pharmacists were included. Non-Malaysian pharmacists were excluded from the study.

Using a written information sheet, participants were notified of the study aim, informed that their participation was voluntary, and that their responses were anonymised. A cover letter was attached to the questionnaire, detailing informed consent, as well as instructions for completing and returning the sheets. The questionnaires were distributed for self-administration by the pharmacists who consented to partake in the study. They were distributed during site visits (68 at district of Klang, 33 at Kuala Selangor, 30 at Sabak Bernam, 22 at Kuala Langat, 23 at Hulu Selangor, 30 in Gombak, 36 in Petaling, 22 in Sepang, and 8 in Hulu Langat). They were each left with a hand-delivered questionnaire, a pen, and a standard return envelope to ensure confidentiality. The face-to-face method of administration had generated high response rates (Samsuri et al., 2015). Approximately 15–20 min were required to complete the survey. Completed questionnaires were sealed in envelopes and none of the data could be traced to any respondent. Attempts were made to contact pharmacists not captured during the site visits. A tracking sheet was used to identify serial numbers from each pharmacy and to track the number of questionnaires given out and those returned. Tracking sheets did not contain any data that could be used to identify a particular respondent. At the onset of the COVID-19 outbreak, the remaining 128 potential respondents were invited *via* email to respond to the questionnaire which was published on Google Docs.

The sample size required was 297 when calculated using the Krejcie and Morgan formula (Krejcie and Morgan, 1970). In order to achieve the minimum sample size required and to give the study sufficient power, 400 community pharmacists were targeted, with a postulated response rate of 50%.

$$s=X^{2}NP\left(1-P\right)÷d^{2}\left(N-1\right)+X^{2}P\left(1-P\right)$$

*s* = required sample size
*X* ^2^ = the table value of chi-square for 1 degree of freedom at the desired confidence level (3.841).
*N* = the population size (1300). Number of community pharmacies registered in Klang Valley in the year 2000
*P* = the population proportion (assumed to be 0.50 since this would provide the maximum sample size).
*d* = the degree of accuracy expressed as a proportion (0.05).

### 2.2 Measures or survey instruments

One of the most recently validated and commonly used tool for measuring current statuses of knowledge, attitudes, perceived concerns and expectations, and willingness of pharmacists to pursue pharmacogenomics, is the 28-item questionnaire by AlEjielat et al. 2016. Since its publication, it had been utilised in several settings worldwide, i.e., among healthcare professionals in Ethiopia and Egypt, (Abdela et al., 2017; Nagy et al., 2020), pharmacy students in Netherlands, (Bank et al., 2018), and pharmacists in Saudi Arabia (Algahtani, 2020) and Thailand (Karuna et al., 2020). Permission to use the questionnaire was granted by the authors.

We used the English version, converted the questions, and made the questionnaire digitally accessible *via* an online platform, Google Docs. This was paramount at the time of the COVID-19 outbreak where face-to-face interviews could not be performed due to a nationwide lockdown. The questionnaire comprised six domains for: 1) respondent demographics, which included gender, age, ethnicity, years of experience, and the highest education qualification, 2) general knowledge, 3) self-assessed knowledge, 4) perceived concerns on practice, 5) past and future training on pharmacogenomics, and 6) willingness to implement in the future. Responses for the knowledge domains were provided as multiple-choice answers with one best answer. Responses for the levels of agreement were presented as a 3- or 5-point Likert scale which ranged from a “strongly disagree” to a “strongly agree” notion (explained further below).

### 2.2.1 Primary endpoints

The research endpoints were: 1) Both acquired and self-assessed knowledge statuses, 2) perception regarding pharmacogenomics, 3) exposure to pharmacogenomics during pharmacy school curricula, 4) willingness of community pharmacists to undergo pharmacogenomics training, and the components of precision medicine training perceived as necessary, 5) pharmacists’ willingness to integrate pharmacogenomics into their current practice.

For endpoint 1, from a multiple-choice answer of “True”, “False”, “I do not know”, respondents who scored 4 points and above were considered to have acquired good knowledge, 3 points moderate, and less than 3 points were considered poor. Using a 3-point Likert scale of “agree”, “disagree”, “neutral”, self-assessed knowledge was deemed good when respondents agreed to at least 4 statements, moderate for 3 statements, and poor when they agreed to 2 statements or fewer. For endpoint 2 on perception, from a 5-point Likert scale (strongly disagree to strongly agree), agree/strongly agree to at least 7 statements was considered good and any fewer was poor. For endpoint 3, exposure to pharmacogenomics during pharmacy education, from a 5-point Likert scale (strongly disagree to strongly agree), agree/strongly agree indicated an exposure. For endpoint 4, willingness to undergo training, from a 5-point Likert scale (strongly disagree to strongly agree), agree/strongly agree indicated willingness. For endpoint 5, from a multiple-choice answer of “yes”, “no”, “not sure”, respondents were considered willing to implement precision medicine when they answered yes to at least 5 statements and the remainder were not willing.

### 2.3 Statistical analyses

The data were analysed using the SPSS^®^ programme, version 23 for Windows^®^ (IBM Corporation, Armonk, New York, United States). Data on the respondents’ characteristics and responses to each statement were presented descriptively as frequencies with percentages. Reverse coding was done for negatively worded statements. As the data were not normally distributed, associations between respondents’ characteristics and the mean ranks of their knowledge, perception, and willingness to practice precision medicine were evaluated using Mann–Whitney *U* and Kruskal–Wallis tests of association. Dunn’s *post-hoc* multiple comparison ANOVA tests with Bonferroni correction were performed following a statistical significance. All statistical tests, except for the *post-hoc* multiple comparison test, were performed at *a priori* significance level of *p* = 0.05. The latter was considered significant at a level of *p* = 0.025.

### 2.4 Ethical consideration

Approval from the University’s Research Ethics Committee (REC) was obtained prior to conducting the study (details below).

## 3 Results

### 3.1 Demographic characteristics

Of the 400 community pharmacists to whom the questionnaires were distributed to (both electronically and face-to-face), 300 completed questionnaires were received (75% response rate). Analysis was performed on the 300 respondents. The characteristics of the respondents are detailed in Table 1.

**TABLE 1:** Sociodemographic characteristics.

Characteristics	N (%)
Gender	
Male	104 (34.7)
Female	196 (65.3)
Age range (years)	
21–30	134 (44.7)
31–40	86 (28.7)
41–50	58 (19.3)
51–60	18 (6)
> 60	4 (1.3)
Ethnicity	
Malay	51 (17)
Chinese	225 (75)
Indian	21 (7)
Others	3 (1)
Years of experience as community pharmacists	
0–10 years	190 (66.3%)
11–20 years	79 (26.3%)
21–30 years	27 (9%)
31–40 years	4 (1.3%)
Highest education qualification	
Bachelor’s Degree	240 (80%)
Master’s Degree	60 (20%)
District of practice	
Gombak	28 (9.3%)
Hulu Langat	50 (16.7%)
Hulu Selangor	21 (7%)
Klang	64 (21.3%)
Kuala Langat	22 (7.3%)
Kuala Selangor	13 (4.3%)
Petaling	64 (21.3%)
Sabak Bernam	11 (3.7%)
Sepang	27 (9%)

More than half of the respondents were females (*N* = 196, 65.3%) and the majority were practising for a mean average of 6.2 years and were grouped in the “category 0.5–10 years” (*N* = 190, 66.3%). A sizeable number of participants were aged 30 years and below (N = 134, 44.7%), had been attached for at least 6 months in a community-based pharmacy registered in any of the nine districts’ postcodes within the state of Selangor. The two areas where a high number of community pharmacists responded to the survey requests were Klang and Petaling districts (*N* = 64, 21.3% each).

### 3.2 Knowledge

#### 3.2.1 General knowledge on precision medicine and pharmacogenomics

A majority (76.3%) possessed moderate levels of knowledge pertaining to the concepts of precision medicine and pharmacogenomics (Table 2). Almost all pharmacists (95%) scored points for the statement “Pharmacogenomics is an integral part of precision medicine and is a combination of both pharmacology and genomic studies,” while many did not know the answer to the statement “Pharmacogenetic testing is currently available for most medications in Malaysia.” The statement, “Genetic determinants of a person towards a drug response can change over a lifetime.” was often misinterpreted.

**TABLE 2:** General knowledge on precision medicine and pharmacogenomics.

Statements on general knowledge	True	False	I do not know
1 Precision medicine describes the prevention, diagnosis and treatment strategies that takes into account differences between individuals	261 (87.0%)	13 (4.3%)	26 (8.7%)
2 Precision medicine only incorporates the variations in a person’s genes while the surrounding environment and the person’s lifestyle does not influence the disease state of a patient	71 (23.7%)	180 (60%)	49 (16.3)
3 Pharmacogenomics is an integral part of precision medicine and is a combination of both pharmacology and genomic studies	285 (95%)	3 (1%)	12 (4%)
4 Pharmacogenetic testing is currently available for most medications in Malaysia	48 (16%)	101 (33.7%)	151 (50.3%)
5 Genetic determinants of a person towards a drug response can change over a lifetime	199 (66.3%)	42 (14%)	59 (19.7%)

#### 3.2.2 Self-assessed knowledge on precision medicine and pharmacogenomics

In general, the pharmacists who self-assessed their level of knowledge were unsure, and did not respond positively or negatively (42.7%, 39%, 36.7%, and 45.3% selected “neutral” to four of the five statements). For the fifth statement that read, “Additional information can easily be obtained online on topics regarding precision medicine and pharmacogenomics”, approximately half were positive (49% selected “agreed”) (Table 3). Overall, slightly more than half (53.7%) of the respondents assessed their self-knowledge as poor, while a proportion (34.7%) assessed that they were moderately knowledgeable, and a small number (11.7%) assessed that they were knowledgeable.

**TABLE 3:** Self-assessment of knowledge on precision medicine and pharmacogenomics.

Statements on self-assessed knowledge	Yes	No	Not sure
1 I know the current pharmacogenetic tests that are currently available in Malaysia	50 (16.7%)	122 (40.7%)	128 (42.7%)
	Agree	Disagree	Neutral
2 I believe that as a community pharmacist, I am well equipped to discuss pharmacogenetic information with other healthcare providers such as doctors, nurses, pharmacists etc.	92 (30.7%)	91 (30.3%)	117 (39%)
3 As a community pharmacist, I can accurately apply the results of a pharmacogenetic test to a current drug therapy, dosing and monitoring of response towards the drug	101 (33.7%)	89 (29.7%)	110 (36.7%)
	Yes	No	Not sure
4 I can identify medications for which pharmacogenetic testing is recommended	84 (28%)	80 (26.7%)	136 (45.3%)
	Agree	Disagree	Neutral
5 Additional information can easily be obtained online on topics regarding precision medicine and pharmacogenomics	147 (49%)	37 (12.3%)	116 (38.7%)

### 3.3 Perception

#### 3.3.1 Perception towards the practice of precision medicine and pharmacogenomics

In general, a majority (94%) demonstrated good perception while a minority (6%) perceived poorly the practice of precision medicine. While many (83.3%) felt that precision medicine had an important part to play in their professional role as a community pharmacist, slightly fewer (80.3%) thought that the use of precision medicine by a community pharmacist will help to decrease the number of adverse effects patients experienced from a drug treatment, and even fewer (72%) expressed that the implementation of precision medicine at the community pharmacy will help improve the efficacy of a drug in a patient. Approximately half (48.4%) were concerned that one of their patient’s pharmacogenetic test results could be passed onto an unauthorised person while a proportion, 57%, were worried that the test results could show that their patients had risk factors for another disease which he/she is unaware of, for example Alzheimer’s and cancer (Table 4).

**TABLE 4:** Perception towards the practice of precision medicine and pharmacogenomics.

Statements assessing perception	Strongly disagree	Disagree	Neutral	Agree	Strongly agree
1 I feel that precision medicine has an important part to play in my profession as a community pharmacist	0 (0%)	4 (1.3%)	46 (15.3%)	196 (65.3%)	54 (18.0%)
2 As a community pharmacist, implementing precision medicine to my profession brings about positive outcomes to my overall practice	0 (0%)	4 (1.3%)	39 (13.0%)	192 (64.0%)	65 (21.7%)
3 Use of precision medicine by a community pharmacist will help to decrease the number of adverse effects patients experience from a drug treatment	0 (0%)	2 (0.7%)	57 (19.0%)	165 (55.0%)	76 (25.3%)
4 Implementation of precision medicine at the community pharmacy will help to improve the efficacy of a drug in a patient	1 (0.3%)	5 (1.7%)	78 (26.0)	149 (49.7%)	67 (22.3%)
5 Pharmacogenomics and precision medicine are fields relevant to my primary care setting	0 (0%)	10 (3.3%)	86 (28.7%)	162 (54.0%)	42 (14.0%)
6 The use of pharmacogenomics and precision medicine in the community pharmacy aids in the optimization of drug dosing	0 (0%)	8 (2.7%)	106 (35.3%)	138 (46.0%)	48 (16.0%)
7 Part of the job scope of a community pharmacist in the area of precision medicine includes counselling patients regarding pharmacogenetic information	0 (0%)	6 (2.0%)	46 (15.3%)	188 (62.7%)	60 (20.0%)
8 Are you worried that, as a community pharmacist, one of your patient’s pharmacogenetic test results could be passed onto an unauthorised person?	8 (2.7%)	49 (16.3%)	98 (32.7%)	98 (32.7%)	47 (15.7%)
9 Are you worried that the test results can show that your patient has risk factors for another disease which he/she is unaware of? (Alzheimer's, cancer etc.)	4 (1.3%)	46 (15.3%)	80 (26.7%)	134 (44.7%)	36 (12.0%)
10 Do you think that a patient’s unfavourable test result may have an adverse psychological consequence on him/her and their family?	5 (1.7%)	34 (11.3%)	97 (32.3%)	128 (42.7%)	36 (12.0%)

### 3.4 Willingness

#### 3.4.1 Willingness to implement precision medicine and pharmacogenomics

While many did concur that as present, they did not counsel patients sufficiently on precision medicine (57.3%), they will be willing to use genome-guided tools (85.7%), willing to offer pharmacogenetic testing (80%), and being a provider of other precision medicine services (88.3%). Time constraints, lack of budget and training were identified as potential barriers to the provision and implementation of such services (Table 5). Nonetheless, 80% of the community pharmacists were willing to practice precision medicine.

**TABLE 5:** Willingness to implement precision medicine and pharmacogenomics.

Statements assessing willingness to implement	Yes	No	Not sure
1 I am willing, as a community pharmacist, to utilise precision medicine, pharmacogenomics as well as genome-guided tools to dispense medication and services to customers	257 (85.7%)	6 (2.0%)	37 (12.3%)
2 As a community pharmacist, I am willing to offer pharmacogenetic testing and counselling as one of my services in the community pharmacy	240 (80.0%)	8 (2.7%)	52 (17.3%)
3 If precision medicine services are implemented in the future, would you like to be a precision medicine service provider?	265 (88.3%)	1 (0.3%)	34 (11.3%)
4 In your current practice as a community pharmacist, do you think that you spend enough time counselling your patient on precision medicine?	69 (23.0%)	172 (57.3%)	59 (19.7%)
5 Do you think you will have enough time as a community pharmacist to apply precision medicine services in the future?	144 (48.0%)	33 (11.0%)	123 (41.0%)
6 Do you usually access any related articles and journals regarding precision medicine and pharmacogenomics?	64 (21.3%)	181 (60.3%)	55 (18.3%)
7 The lack of training is one of the potential barriers regarding the application of precision medicine in the community setting in the future	266 (88.7%)	3 (1.0%)	31 (10.3%)
8 Do you think that applying precision medicine services in the community pharmacy setting would require a high budget?	163 (54.3%)	43 (14.3%)	94 (31.3%)

### 3.5 Prior exposure

#### 3.5.1 Exposure to pharmacogenomics in pharmacy school curricula, willingness of community pharmacists to undergo pharmacogenomic training, and the components of precision medicine perceived necessary

A high proportion of the community pharmacists did not or do not recall having a prior exposure to pharmacogenomics as part of their pharmacy school curricula (61%). Nonetheless, a huge proportion (93%) were willing to acquire knowledge on precision medicine by undergoing additional training (Table 6).

**TABLE 6:** Exposure to pharmacogenomics in pharmacy school curricula, willingness of community pharmacists to undergo pharmacogenomics training.

I had training on precision medicine and pharmacogenomics as part of the pharmacy curriculum			
Likert scale	Frequency	Percent	Cumulative percent
Strongly disagree	19	6.3	6.3
Disagree	67	22.3	28.7
Neutral	97	32.3	61.0
Agree	100	33.3	94.3
Strongly agree	17	5.7	100.0
Total	300	100	100
I am willing to undergo training to acquire knowledge on precision medicine and pharmacogenomics			
Strongly disagree	0	0	0.0
Disagree	0	0	0.0
Neutral	21	7.0	7.0
Agree	191	63.7	70.7
Strongly agree	88	29.3	100
Total	300	100.0	100

**FIGURE 1:**
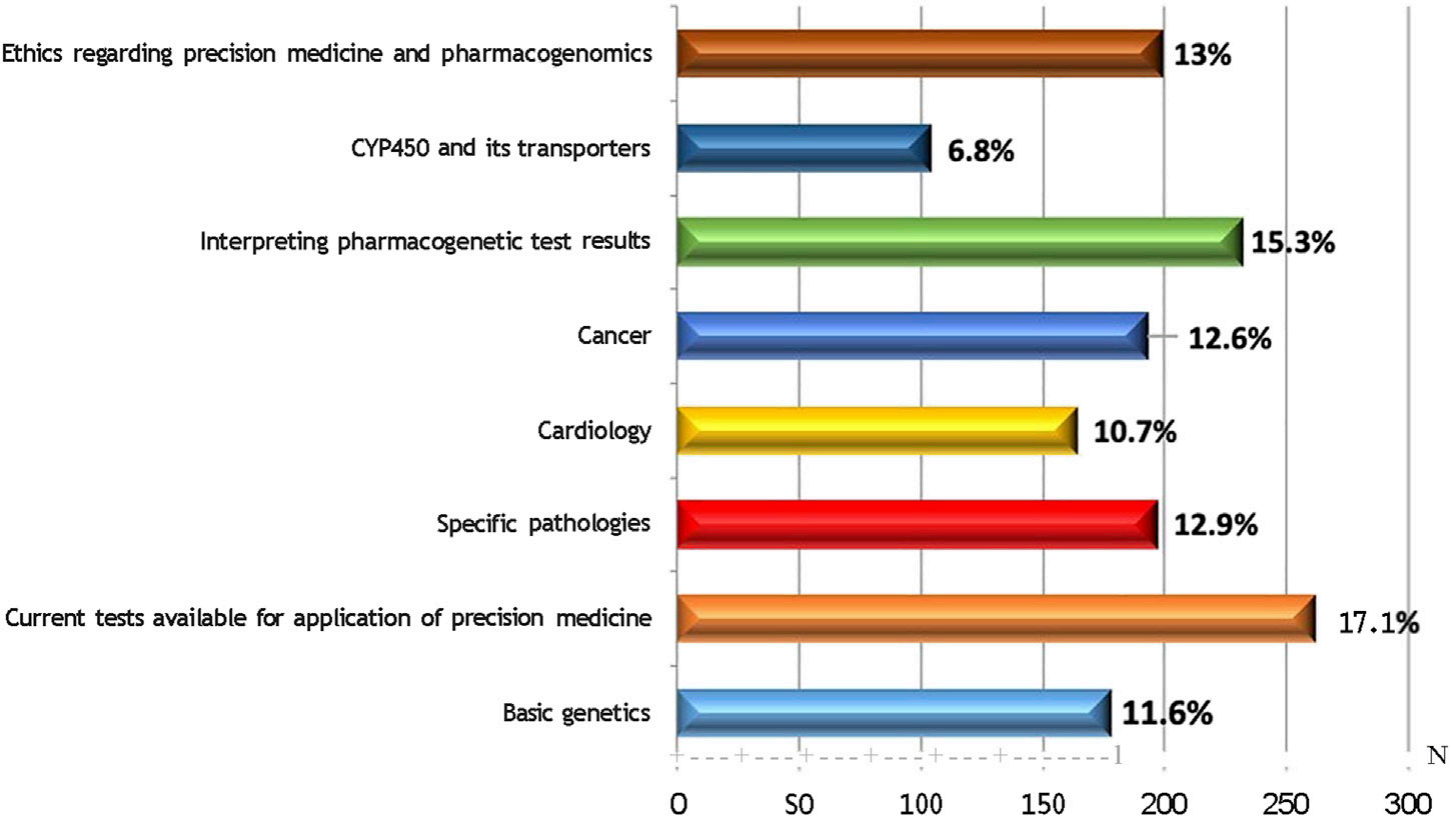
Components of Precision Medicine Perceived Necessary to be Trained On.

Among the multiple-choice of eight components of precision medicine offered to the community pharmacists for the selection of one that they would prioritise to undergo training on, most had selected to undergo tutoring on the current pharmacogenetic tests that were accessible (17.1%), followed by guidance on interpreting the test results (15.2%), and subsequently the ethical aspects regarding precision medicine. Learning about the cytochrome P450 (CYP450) enzyme and its transporters was the least favoured component, 6.8% (Figure 1).

### 3.6 Differences in knowledge, perception and willingness to practice

While there were statistically no significant differences between any sociodemographic characteristic and perception or willingness to practice, good general knowledge on precision medicine was significantly associated with the community pharmacists’ years of work experience [H(3), *p* = 0.004]. Further, multiple ANOVA tests for total knowledge scores amongst categories “0.5–10 years”, “11–20 years”, and “21–40 years” revealed significance [F (2, 195) = 4.146 *p* = 0.045]. Post hoc comparisons using Bonferroni correction at 0.025 level of significance, revealed a significant difference between the community pharmacists who had 0.5–10 years’ work experience and expressed greater acquired knowledge (μ = 1.48, CI 1.35–1.61, *p* = 0.017), than the pharmacists who had 21–40 years’ of work experience (μ = 1.28, CI 1.05–1.51, *p* = 0.021). No statistically significant difference existed between the other work experience groups.

### 3.7 Differences in willingness to practice, and prior exposure to pharmacogenomics as part of pharmacy education.

Community pharmacists who had exposure to pharmacogenomics as part of their pharmacy curricula were more inclined and willing to integrate precision medicine in their daily practices as compared to those who did not, or who were unable to recall (“neutral”) having had a similar exposure (mean rank = 214.3, *U* = 337.5, *p* = 0.038).

## 4 Discussion

### 4.1 Survey response rate

The method of hand-delivering a questionnaire, mixed with distribution of the digital content to individual emails during the early COVID-19 pandemic, generated a moderately high response rates (75%). In particular, the “email survey” approach, which involved sending the questionnaire as an attachment to an email as opposed to having a “hyperlink embedded” in the email was useful for obtaining responses. Telephone calls or postal mails have been conventionally used. However, in recent times, particularly in the past two years, there has been an upsurge trend in the use of web surveys (Sammut et al., 2021).

### 4.2 Barriers to implement precision medicine

The study revealed that there is much room for improvement on the levels of knowledge, and is possibly an indicator that precision medicine in low and middle income countries are very much at its infancy (Haque et al., 2020). Similar to the findings of this study, where pharmacists had good perceptions and were willing to pursue precision medicine (albeit in moderation), past research inferred that the idea of precision medicine, as well as pharmacogenomics, had been well received among primary care providers including general practitioners and pharmacists (Luke et al., 2021; Surofchy et al., 2021; Gammal et al., 2022). In general though, the endorsement of pharmacogenomics at that time was discouraging, but the prospect of adopting it in the future, garnered much attention (Wicclair, 2011; Bannur et al., 2014). It was observed that the lack of information and skills among doctors and other medical professionals were the main setbacks in clinical utilisation of precision medicine and pharmacogenomics. This was reiterated in the present study where the community pharmacists were aware of their lack of knowledge and were honest to self-assess their level as poor. Similar findings were recently revealed in Nigeria and in Thailand (Karuna et al., 2020; Abubakar et al., 2022). The more prominent countries such as China, Japan, India, or Middle Eastern countries including Qatar, Saudi Arabia and Kuwait have all been more open to the implementation of precision medicine in their clinical care system (Bagher et al., 2021; Arafah et al., 2022).

One of the reasons for hesitancy among low and middle income countries is that precision medicine requires additional expenditure for testing and as healthcare budgets are restricted, effective delivery of healthcare based on pharmacogenetic testing is unpopular (Rahim et al., 2014). A study conducted by the National University of Malaysia, highlighted the challenges faced by Malaysia in introducing the concept of precision medicine into the healthcare sector. For a middle-income developing nation, the expenditure for genetic testing is high and can fetch up to MYR2000-RM5000 (up to USD 1130) for genetic testing and genotyping which is not funded by the nation’s healthcare system. Besides that, tyrosine kinase inhibitors which are touted to be the main treatment option for precision medicine are not listed in the Ministry of Health’s formulary. This would imply that the patients themselves would need to spend their own money, hence incurring extra cost, and burdening the patients. Finally, the eligibility of patients to acquire health insurance coverage may be dampened in lieu of the pharmacogenetic test results. Although Malaysians are not subjected to such laws and regulations, other nations have such ruling where genetic information may be accessed by insurance providers.

In the present study, pharmacists who had 0.5–10 years of experience in the community field had better comprehension of precision medicine as compared to pharmacists who had worked even longer. This is probably because experiences and lessons learnt during their study years were heightened and better understood during their early career years. Similar inferences were made in a study conducted in Romania by Pop et al. 2022. A recent survey of the Southeast Asian Pharmacogenomics Research Network (SEAPHARM) showed that, in Malaysia, contents for basic genetic and pharmacogenomics are integrated within the national pharmacy curriculum. In addition, various platforms are available for continuing education support such as post-graduate training courses, conferences, workshops and online resources for pharmacogenomics (Chumnumwat et al., 2019). Needless to say, the disparities in knowledge gaps among community pharmacists is still prevalent and may be a possible indicator for the low knowledge scores (Macdonald and Yu, 2011).

In general, the pharmacists disagreed or were neutral about their ability to discuss pharmacogenetic information with other healthcare professionals or apply pharmacogenetic test results to current drug therapy possibly because they felt unequipped. Therefore, a supportive environment for continuing pharmacogenomics education aimed at enhancing awareness and strengthening knowledge of community pharmacists in Malaysia, could make its implementation a reality in the near future.

### 4.3 Role of precision medicine and pharmacogenomics in pharmacy school curriculum and willingness of community pharmacists to undergo training on precision medicine and pharmacogenomics

One of the main causes of limited understanding of precision medicine and pharmacogenomics may be the absence of exposure to precision medicine and pharmacogenomics during their pharmacy school years. The culmination of this study showed that a sizeable number of respondents had received some form of education and knowledge on precision medicine and pharmacogenomics during their formative years in pharmacy school. This impacted their receptiveness and willingness to pursue this field further. McMahon 2011 intended to analyse the receptiveness of Australian pharmacists towards the idea of pharmacogenetics. The general view was that those pharmacists were lacking in knowledge about pharmacogenetics and the study revealed that recent pharmacy schools leavers had a better comprehension as compared to longer practicing pharmacists. Interest among pharmacy students in implementing pharmacogenomic testing in clinical practice was low in Saudi Arabia. There was a need to introduce an up-to-date curriculum for pharmacy courses incorporating pharmacogenomics-based health education programs in Saudi Arabia.

One of the primary roles of community pharmacists in the field of pharmacogenomics is fostering the optimal use of pharmacogenomic tests, and elucidating the clinical implications of the test results to other pharmacists, healthcare professionals, patients as well as the general public (Luke et al., 2021). The community pharmacists in our study were inclined to offer pharmacogenetic testing and counselling as part of value added services in their pharmacy and were keen to become a precision medicine provider in the near future. From our survey, the two areas they were willing to train on were the current pharmacogenetic tests available for use for and the interpretation of the pharmacogenetic test results. Ethical issues regarding precision medicine and pharmacogenomics followed suit. Information on cytochrome P450 enzymes and its transporters were the least favourite area among the pharmacists.

In the era of outcome-based education, backward design is a framework for identifying required content or activities of the courses, conferences, webinars, or workshops to help graduates or learners achieve desired outcomes or competencies. In pharmacogenomics, several sets of competencies have been developed (Rahma et al., 2021; Gammal et al., 2022). For example, the framework and core competencies by the American Association of Colleges of Pharmacy (AACP) are systematically organized into two major categories: foundational genetics concepts, and clinical pharmacogenomics (CP), which is constructed based on 6 domains of entrust able professional activities (EPAs) involving: 1) patient care provider, 2) interprofessional team member, 3) population health promoter, 4) information master, 5) practice manager, and 6) self-developer (Kanmaz et al., 2020). These competencies can be utilized as outcomes for the pharmacy curriculum or continuing education activities, although several of the activities in the EPA domains are specific to the pharmacy systems in the USA. Tailoring outcomes to match the ongoing issues contextual to an individual country, its concerns, misperceptions, or specific needs, would help prioritize and optimize contents on, for instance, health and reimbursement policies, or available pharmacogenetic tests or technologies. In this way, pharmacogenomics education programme for pharmacy students, and community pharmacists who are eager to pursue additional pharmacogenomic training, like for the pharmacists in this study, can be effectively developed.

## 5 Strengths and limitations

Due to purposive sampling, the research was aimed at community pharmacists practising in a developed state with the largest economy in Malaysia. Although the response rate was moderately high, and the researchers surveyed areas which were densely populated with community pharmacies, the sample of community pharmacists may not necessarily be nationally representative. Hence, the inferences made from the study findings may only have significant implications to the practitioners local to that area. For instance, pharmacists in less affluent areas may view pharmacogenomics and its related services as a burden. For future research, additional steps need to be taken through improved survey procedures such as, performing a systematic random sampling, to cover a broader population which will then be a more reasonable representation of the country.

## 6 Conclusion

Community pharmacists were receptive to and valued precision medicine. A relatively high number had prior exposure to concepts through the pharmacy curriculum, and were therefore very willing to adopt and implement precision medicine in their day-to-day practice. With adequate training centred on bioethics, utilising pharmacogenetic testing, and interpretation of the results, community pharmacists will be equipped for the provision of precision medicine services in the foreseeable future. In this sense, professional and continuous development programmes focused on structured skill-based learning are likely to successfully inculcate precision medicine among pharmacists.

## Data Availability

The original contributions presented in the study are included in the article/supplementary material, further inquiries can be directed to the corresponding author.

## Abbreviations

EHR, electronic health records; HGP, human genome project; KAP, knowledge, attitude and practice; LMIC, low middle-income countries; NCD, non-communicable diseases; PGx, pharmacogenomics; PM, precision medicine.
